# Single nucleotide polymorphisms on Cholecystokinin B Receptor gene as a candidate gene for crowing in Pelung chickens

**DOI:** 10.5455/javar.2025.l881

**Published:** 2025-03-24

**Authors:** Indrawati Yudha Asmara, Nena Hilmia, Dani Garnida

**Affiliations:** Department of Animal Production, Faculty of Animal Husbandry, Universitas Padjadjaran, Sumedang, Indonesia

**Keywords:** CCKBR, non-synonymous mutation, Pelung chickens

## Abstract

**Objective:**

This study aims to explore mutation based on single nucleotide polymorphism (SNP) in the Cholecystokinin B receptor (CCKBR) gene of Pelung chickens.

**Materials and Methods:**

We collected DNA samples from 48 Pelung roosters that had won the crowing competition. The CCKBR target encompasses exon 3, intron 3, exon 4, and a part of intron 4, a long 601 bp. This target was replicated using PCR with specific primers that were designed by Primer-BLAST from NCBI. We generated the nucleotide sequence from the PCR product’s sequencing results. The SNP analysis was done by BioEdit and MEGA. Genotyping and haplotyping were done based on nonsynonymous single nucleotide polymorphisms (SNPs) on exons 3 and 4. We calculated allele and genotype frequency, heterozygosity, and Hardy-Weinberg Equilibrium (HWE) using POPGENE 32 programs.

**Results:**

This study found three nonsynonymous single nucleotide polymorphisms. The nsSNP in exon 3 alters the coding for the 210th amino acid from serine to asparagine (g.1290 G > A/S210N), while the SNPs in exon 4 alter the coding for the 232nd amino acid from valine to phenylalanine (g.1423G > T/V232F) and the 243rd amino acid that changes the amino acid valine to glycine (g.1457T > G/V243G). The frequency of the mutated alleles is lower than the unmutated alleles. However, the mutation at position g.1457T > G/V243G produces a higher frequency than the unmutated allele. The allele and genotype frequency were not in HWE. It was caused by intensive selection in Pelung chickens, especially for growing capacity.

**Conclusion:**

Nonsynonymous mutation on CCKBR may cause variations in the crowing and other traits such as the growth of Pelung chickens. Further studies are needed to explore the CCKBR gene, including the relationship of the gene with the vigor and/or stress level of Pelung chickens

## Introduction

In Indonesia, people keep Pelung chickens, among other local chickens, for their long-crowing capacity [1]. In addition to their long, distinctive, and melodious crowing, the posture of Pelung chickens is taller and bigger than that of other local chickens. Some studies indicated that Pelung chickens have potential as meat producers [2]. Due to their unique crowing characteristics, enthusiasts carry out Pelung contests regularly. In this event, an exchange of information among breeders and enthusiasts about the rearing technique of Pelung breeds and trading transactions may occur. This event becomes a supporting system for the existence of Pelung chickens.

Chickens and other birds produce vocalizations classified as songs and calls. Crowing is part of the song category. Unlike songbirds that can learn vocalizations through imitation, chickens can only produce specific vocalization patterns according to their species, called innate vocalization. Genetic factors could likely influence crowing [3]. This conclusion is in line with breeders who believe that hereditary factors influence the crowing ability of Pelung roosters [4,5]. This argument is proven by the fact that a crowing contest winner would be used as a breed, and their selling price would be valued higher. In addition, the age of roosters may influence the crowing capacity of Pelung. Older roosters would have longer crowing than younger ones [5]. Crowing only occurs in roosters, not in hens; thus, it is known as a testosterone-dependent behavior [3]. Due to having optimum testosterone production, older Pelung roosters would have more stable crowing ability [5].

As a qualitative trait, the crowing ability of chickens is normally induced by a limited number of genes. One of the potential candidate genes that influence crowing ability in birds is the Cholecystokinin B Receptor (CCKBR) gene. Cholecystokinin (CCK) is a neurotransmitter that is commonly expressed in the brains of mammals and birds. The highest expression of CCK is in the hypothalamus of chickens, and it is associated with behavior regulation such as anxiety, learning, and memory [6]. As a neurotransmitter, CCK binds CCKBR. The expression of CCKBR depends on androgens, which accelerate the binding of CCK to CCKBR. This mechanism is supported by the fact that chicks induced by testosterone can do crowing and the expression of CCKBR is higher in roosters [3].

The detection of single nucleotide polymorphisms (SNPs) can facilitate the exploration of candidate genes [7]. We commonly use SNPs, the preferred molecular genetic markers, to test for relationships between phenotypes and genetic variation [8]. This study proposes to identify SNPs in the CCKBR gene as a candidate gene for crowing in Pelung chickens. The SNPs may contribute to crowing capacity, variation, and other economic traits. It can be used as marked-assisted selection in crowing chickens to support effective and efficient genetic selection.

The unique ability of birds to make sounds is a quality trait that is mostly controlled by a small group of genetic factors. Empirical investigations have identified several genes contributing to bird vocalization. Nevertheless, research to clarify the genetic components associated with the crowing phenomenon in chickens remains scarce. This study intends to bridge the existing knowledge gap concerning potential candidate genes implicated in the crowing behavior of chickens, with a particular focus on Pelung chickens. To the authors’ knowledge, this investigation marks the introductory effort to examine CCKBR as a prospective candidate gene for crowing in Pelung chickens.

## Materials and Methods

### Ethical approval

The Research Ethics Committee at Universitas Padjadjaran Bandung, Indonesia, has approved the research protocol under approval number 218/UN6.KEP/EC/2024.

### Sample collection

**Figure 1.**
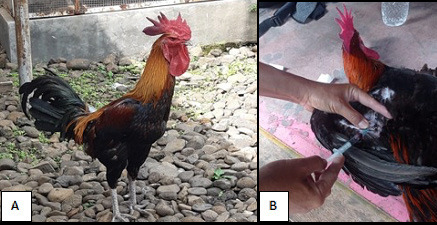
A) Male Pelung chicken, (B) Blood collection from Pelung chickens.

The purposive sampling method selected Pelung roosters as the samples. The 48 roosters that prevailed in the crowing competition served as a sample criterion. Pelung keepers were asked for their willingness to provide their roosters as samples through an oral consent form. A trained investigator was involved in collecting blood samples (Fig. 1).

### Procedures

#### DNA extraction

We took blood samples from the vein brachialis using a syringe (3 ccs) and then stored them in a Vacutainer with EDTA anticoagulant. The DNA is isolated using the Zymo Research Quick-DNATM Miniprep Plus Kit. The DNA isolation results were checked with 1.2% agarose gel electrophoresis at 100 V for about 60 minutes, and the gel was seen with an ultraviolet (UV) transilluminator (Gel Doc).

#### CCKBR gene amplification

PCR is a technique of duplicating a target DNA molecule flanked by a pair of primers. Amplification of the target gene used Master Mix PCR Thermo 14001012 Invitrogen Platinum II Hot Start Green. Each PCR reagent was made into a volume of 25 µl with the composition of 50 ng template DNA, 0.25 µM each of forward and reverse primers, and PCR master mix. The primers used are Forward Primer 5’-CTG CCT GTG ACC GTA CAC C-3’ and Reverse Primer 5’-CGT TTC CCA GAG CCT CAC CT-3’. Primers were designed using Primer BLAST NCBI. The target of the CCKBR gene is 601 bp, comprising a part of exon 3, intron 3, exon 4, and a part of intron 4. The PCR machine, which is used by LongGene Scientific Instruments, Model A600, serial: 016-00357.

Amplification began with one initial denaturation at 94°C for 4 min, followed by 35 times denaturation at 94°C for 45 sec, annealing at 59°C for 1 min, and extension at 72°C for 1 min. A further extension was carried out at 72°C for 5 min. The PCR products were evaluated using 1.2% agarose electrophoresis and visualized with a UV transilluminator for 60 min.

### Data analysis

The SNPs in the CCKBR gene were analyzed based on direct sequencing results. The sequencing results were aligned using the BioEdit and MEGA X programs. Genotyping and haplotype were analyzed based on polymorphism (SNPs) in exon 3 (20 amino acids) and exon 4 (43 amino acids). Genotype and allele frequency, heterozygosity, and Hardy-Weinberg Equilibrium were calculated by the POPGEN32 program.

## Results

**Figure 2.**
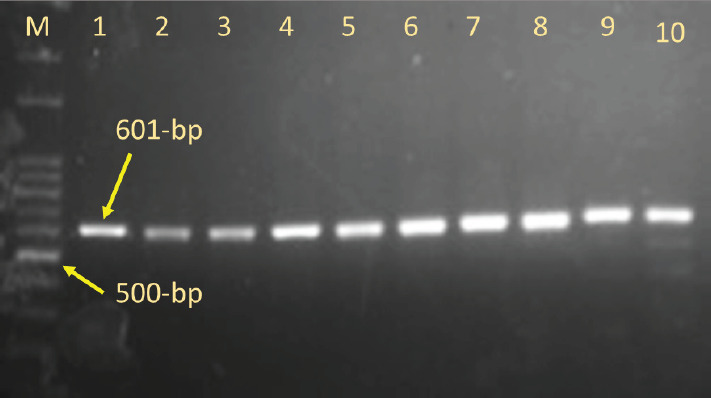
PCR amplification of CCKBR gene. M = ladder, Lanes 1 to 10 samples (601 bp).

The target nucleotide sequence of the Pelung chicken CCKBR gene was amplified using primers that can amplify the nucleotide sequence of the CCKBR gene, encompassing part of exon 3, intron 3, and exon 4, a long 601 bp (Fig. 2). Point mutation on the nucleotide sequence of CCKBR gene target SNPs was analyzed from PCR product sequencing results.

**Figure 3.**
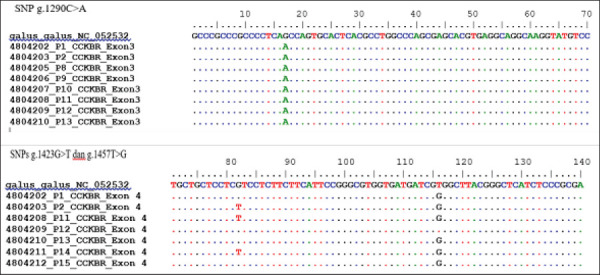
Single nucleotide polymorphism g.1290C > A in exon 3 and SNP g.1423G > T and g.1457T > G in exon 4.

The result was analyzed and confirmed with nucleotide sequence data on the CCKBR gene in chickens (Gallus) from the Gene Bank NCBI with access code NC_052532.1. The alignment result of the CCKBR sequence found three nsSNPs; one nsSNP, g.1290C > A, was found in exon 3 and two nsSNPs in exon 4, namely g.1423G > T and g.1457T > G (Fig. 3).

**Figure 4.**
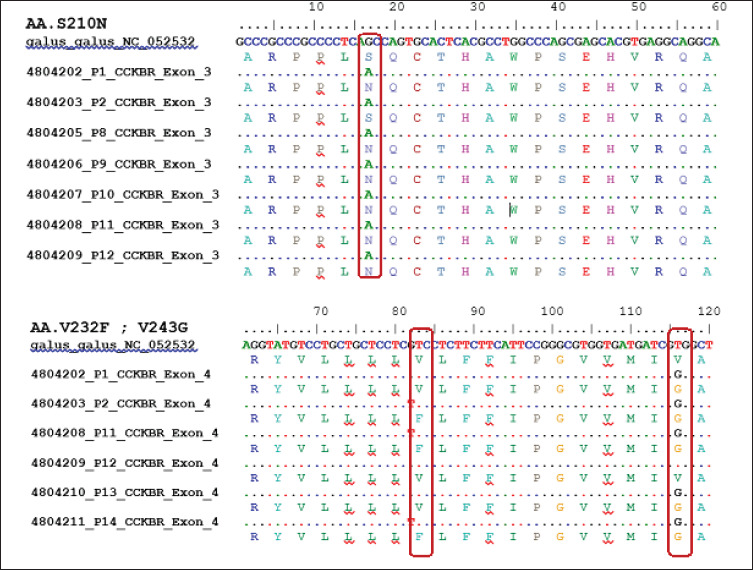
Nonsynonymous SNPs S210N (g.1290C > A) on Exon 3 and V232F and V243G (g.1423G > T and g.1457T > G) on Exon 4 CCKBR gene of Pelung chickens.

The current study found three nsSNPs that alter amino acid coding. The nsSNP in exon 3 alters the coding for the 210th amino acid from serine to asparagine (S210N), while the SNPs in exon 4 alter the coding for the 232nd amino acid from valine to phenylalanine (V232F) and the 243rd amino acid that changes the amino acid valine to glycine (V243G) (Table 1). The alignment results of altering amino acids are presented in Figure 4.

In addition, this study found three nucleotide mutations SNPs at the intron positions of the nucleotide sequences C1369T, G1381T, and C1395T (Access No. NC_052532.1). Mutations at the three positions occurred by substitution or replacement of the nitrogen base from C (cytosine) or G (guanine) to T (thymine). Mutations in introns, which are non-coding areas, do not directly change the coding of amino acids in forming proteins that form hormones or enzymes. Some literature states that mutations in introns will affect the transcription process. Based on this argument, we genotyped the study using nsSNPs on the exon segment (Table 2).

**Table 1.** The SNPs, type of mutation and amino acids changes of CCKBR gene in Pelung chickens.

No	SNP	Type of mutation	Amino acid changes
1	g.1290 G > A/ S210N	nonsynonymous	Serin to Asparagine
2	g.1423G > T/V232F	nonsynonymous	Valin to Phenylalanine
3	g.1457T > G/V243G	nonsynonymous	Valin to Glycine

Based on genotyping results in the coding area of Exon 3 g.1290 G > A and Exon 4 g.1423 G > T), the frequency of the mutated allele is lower than the unmutated allele; however, the mutation at position g.1457T > G produces a higher frequency. Based on three nonsynonymous mutations, five haplotypes can be developed (Table 3). The haplotype based on three nonsynonymous SNPs in the present study reveals haplotype 5 types higher than that of other haplotypes. Haplotype 5 consists of a non-mutation sequence in exon 3 and exon 4. A lower frequency in this study was found in haplotype 4, which just has one mutation on g.1457T > G/V243G.

## Discussion

Birds emit sounds that fall into two categories: calls and songs. The song is a long and complex pattern of sounds, and crowing is an example of a call rather than a song. Long-crowing chickens crow longer than normal chickens. The long-crowing chicken is commonly found in Asia, including Indonesia. The long-crowing chickens in Indonesia are Pelung chickens from West Java Province, Kokok balenggek from West Sumatra Province, Bekisar from East Java Province, and Gaga or Ketawa chickens from South Sulawesi Province. Studies have found genes like FOXP2, Zenk, Dopamine Receptor, and Cholecystokinin B Receptor that affect a bird’s ability to sing, even in chickens [1].

It is the CCK gene’s receptors that make it work. Cholecystokinin is the most common neuropeptide in vertebrate brain peptide hormone. CCKAR and CCKBR are individually functional in peripheral organs and the central nervous system. There is not much CCKAR expression in the brain, but there is a lot of CCKBR expression in the brain and the rest of the central nervous system (CNS) [9]. In chickens, the CCKBR neurotransmitter is commonly expressed in different parts of the CNS, such as the cerebrum, midbrain, and hindbrain. Furthermore, the CCKBR is a controlling gene in the crowing of roosters [7]. The study looked at how much CCKBR is expressed in the nucleus intercollicularis (ICo) of the midbrain. This is because the ICo is thought to be where rooster crowing is concentrated. A mutation refers to a change in the nucleotide sequence within the coding regions of DNA, which may alter the amino acid sequences of proteins. On the other hand, a mutation can happen in the non-coding parts of DNA, which could change gene expression by changing things like how strong a promoter is [10]. The current study found three nsSNPs in the CCKBR gene of Pelung chickens. One nsSNP was found in exon 3 and the other two were found in exon 4. A nonsynonymous mutation changes the corresponding amino acid in the protein [11,12].

**Table 2.** Genotype, allele frequency, heterozygosity and hardy weinberg equilibrium of CCKBR gene in Pelung chickens.

Mutation/SNP	Genotype frequency			Allele frequency		Ho	He	*x*^2^ value	*p* value
g.1290 G > A	GG	GA	AA	G	A				
S210N	0.500	0.042	0.458	0.521	0.479	0.042	0.504	41.259	0.000
g.1423G > T	GG	TG	TT	G	T				
V232F	0.875	0.125	0	0.938	0.062	0.125	0.117	0.176	0.675
g.1457T > G	TT	TG	GG	T	G				
V243G	0.542	0.229	0.229	0.656	0.344	0.229	0.451	12.183	0.000

Note: Ho= observed heterozygosity; He= expected heterozygosity; *p* value (*p* < 0.05).

Based on the results (Table 1), two SNPs are categorized as transition mutations, and one SNP is a transversion. A transition mutation changes a purine base to a pyrimidine base or a pyrimidine base to a purine base [13]. A transversion mutation changes a purine base to a pyrimidine base or the other way around [13]. Transversion mutations have big effects on regulatory DNA, like looking at transcription factor (TF) binding motifs and TF binding that is specific to an allele. These mutations are more likely to disrupt TF binding, leading to substantial changes in gene expression [14].

**Table 3.** Haplotype polymorphisms of CCKBR Gene in Pelung chickens.

Haplotype	g.1290 G > A	g.1423G > T	g.1457T > G	Frequency (%)
Haplotype 1	A	G	G	18.86
Haplotype 2	A	G	T	18.86
Haplotype 3	A	T	G	11.32
Haplotype 4	G	G	G	5.66
Haplotype 5	G	G	T	45.28

In this study, haplotype construction included nonsynonymous mutations. The construction process focused on mutations within a set of simultaneously inherited genomes. A haplotype represents a group of DNA variants arranged along a single chromosome and typically inherited together. This grouping occurred due to their proximity on the chromosome, resulting in rare recombination events between these variants. Haplotypes could vary in size, from a single gene to encompassing multiple genes. Haplotype diversity is shaped by various factors, including mutation, recombination, marker selection, and demographic influences [15].

It is less likely for nonsynonymous mutations to happen than synonymous mutations [11]. This is because natural selection, especially purifying selection [12], tends to target nonsynonymous mutations more often. In livestock populations, a nonsynonymous variation characterizes raw material for selection to adapt to various environmental circumstances [16]. In nsSNPs, the minor allele frequency is likely lower [17]. The most impactful allele variations caused by SNPs are those that influence protein stability because they can interrupt all protein interactions [18]. Significant changes in amino acids reveal important sites for maintaining protein structure [17]. According to a study by Beinfield [19], when the amino acid His207 is taken away from the CCKBR receptor, CCK cannot bind to it. The way that CCK binds to its receptor changes when the positions of the amino acids His207 and asparagine are switched around. The current study revealed that the frequency of the mutated allele is lower than that of the unmutated allele. However, we also identified a high allele frequency for g.1290 G > A (A allele = 0.479). It could be due to the intensive selection of superior characteristics possessed by Pelung chickens.

Heterozygosity reflects substantial genetic variability in the population; it is high if there is wide variation in genetic capacity, while low heterozygosity reflects limited genetic variability. In this study, two SNPs, g.1290 G > A and g.1457 T > G, have lower observed heterozygosity compared to expected heterozygosity, suggesting that selection or inbreeding has taken place in Pelung chicken populations. When observed heterozygosity is lower than expected, it indicates inbreeding [20]. Additionally, a chi-square test showed that the SNPs g.1290 G > A and g.1457 T > G are not in Hardy-Weinberg equilibrium, further indicating inbreeding or selection in the Pelung chicken population. A population is in Hardy-Weinberg equilibrium when there is no mutation, random mating occurs, the population size is large, and there is no natural selection [21]. The selection process in Pelung chickens focuses on crowing capacity. Hardy-Weinberg equilibrium can be upset by a lot of things, such as genotyping mistakes, purifying selection, copy number changes, inbreeding, and population substructure [22,23]. Núñez Dominguez [24] reported that the Mexican Romosinuano population has been genetically isolated due to sanitary regulations that prevent the import of genetic material from Colombia. Additionally, breeder selection practices and the population’s limited genetic diversity have also contributed to these deviations.

The selection of Pelung is usually based on the character of the crowing sound. An alteration in the amino acid sequence can lead to changes in the folding and stability of the protein, its interactions with other molecules, its functional levels, or its overall function. While a mutation in the amino acid sequence may change the protein’s structure, it does not always affect its function. On the other hand, mutations at certain locations, like conserved residues, change the structure and function of the protein in big ways [25]. Especially mutations in CCKBR may cause variations in the crowing of Pelung chickens.

A study revealed that nsSNPs may cause detrimental effects, such as causing diseases, frequently by decreasing the stability of the protein [18]. Breeders and enthusiasts of Pelung chickens normally do not carry out contests during disease seasons, especially during the seasonal transition period in which avian diseases such as Newcastle disease appear. Pelung roosters, who regularly compete in championships, often exhibit a shorter lifespan. This fact implies that vigor is an important issue for Pelung chickens. Singing would increase the metabolism rate, and a compensatory process to singing by reducing other components of metabolism is likely to occur [26].

In addition, crowing activity may indicate a stress level. A study reported that crowing in chickens is also provoked by social stressors, such as when an unfamiliar individual crows [27]. The findings may verify that social pressure causes anxiety by increasing the CCKBR hormone, which can induce crowing sounds in chickens. We need to conduct further studies to investigate the correlation between vigor, stress levels, and the variation of the CCKBR gene in Pelung chickens.

This study has demonstrated there were nsSNPs of the CCKBR gene as a candidate gene for the crowing sound of chickens. A previous study that characterizes the expression of CCK in chickens indicates the possible involvement of this gene in song production [3]. The existence of nsSNP mutations in the CCKBR gene in Pelung chickens is an initial study to explore candidate genes, especially for crowing capacity variation in these chickens. The mutation in the DNA sequence contributes to variation in gene expression, which can impact their function in the physiological process. The variability of nsSNPs may influence the protein’s structural and functional attributes [18]. nsSNPs located on a protein’s surface are often better tolerated, especially when the side chain faces away from the protein and into the solvent, allowing for potential accommodation of other side chains. However, if the nsSNPs occur in a crucial functional region on the protein’s surface, it could disrupt its function. When nsSNPs alter an amino acid involved in a protein-protein interface, it has the potential to hinder the interaction between proteins [17].

Mutation of the CCKBR gene in Pelung chickens may alter their expression in the brain, which can impact the physiological process of the CNS. Altering hormones in the CNS, especially CCK and their receptor CCKBR, may contribute to a variation in innate vocalization, including crowing capacity. This research is preliminary research to determine the involvement of genes that influence the crowing ability of Pelung chickens, which are thought to be inherited from parents to their offspring. This is in line with the conditions in Pelung chicken breeders, who carry out selection for crowing capacity based on heredity, not based on the ability to imitate as are the songbirds.

Besides having something to do with crowing, SNPs in the CCKBR gene are important for studying differences in traits that are affected by the CCKBR hormone, like growth traits. Pelung is recognized for its fast-growing trait, causing a bigger posture compared to other local chickens. Studies report the mechanism of cholecystokinin (CCK) and CCKBR as its receptor in gastrointestinal functions in fish [28,29] and chickens [6,30]. These studies show that CCK and its receptors play a significant role in the digestive system and have an effect on the growth of living things.

The current investigation involved a sample size of 48 chickens, which may not accurately reflect the larger population of these chicken breeds. An increased sample size has the potential to yield findings that are more robust and widely applicable. In addition, the study did not thoroughly examine environmental variables such as nutrition, living conditions, and breeding, which could also have an impact on findings. Future inquiries might also investigate the environmental determinants that shape chicken crowing characteristics.

## Conclusion

The current study is an initial exploration to determine CCKBR as a gene candidate for crowing in Pelung chickens. This study found three nsSNPs, which alter amino acid coding. Nonsynonymous mutation on CCKBR may cause variations in the crowing and other traits such as the growth of Pelung chickens. We need to conduct further studies to investigate the CCKBR gene and its correlation with the vigor and stress levels of Pelung chickens. Pelung chickens are animal genetic resources in Indonesia that need comprehensive conservation. The uniqueness of their crowing sets them apart from other areas in Indonesia and other countries. Due to their uniqueness, Pelung chickens have significant contributions to the social, cultural, and economic lives of communities, including the keepers and enthusiasts. This study is among the first to explore gene candidates for crowing in Pelung chickens. Thus, the finding is crucial as input for Pelung conservation to support effective and efficient genetic selection.

## List of abbreviations

CCK, cholecystokinin; CKKAR, cholecystokinin a receptor; CNS, central nervous system; DNA, deoxyribonucleic acid; HWE, hardy weinberg equilibrium; ICO, intercollicularis; ND, Newcastle disease; nsSNPs, nonsynonymous single nucleotide polymorphisms; PCR, polymerase chain reaction; SNPs, single nucleotide polymorphisms.
